# Mango (*Mangifera indica* L.) Polyphenols: Anti-Inflammatory Intestinal Microbial Health Benefits, and Associated Mechanisms of Actions

**DOI:** 10.3390/molecules26092732

**Published:** 2021-05-06

**Authors:** Hyemee Kim, Maria Joselyn Castellon-Chicas, Shirley Arbizu, Stephen T. Talcott, Nicholas L. Drury, Shayna Smith, Susanne U. Mertens-Talcott

**Affiliations:** 1 Department of Food Science and Nutrition, Pusan National University, Busan 46241, Korea; 2 Department of Food Science and Technology, Texas A&M University, College Station, TX 77843, USA; m.joselyn@tamu.edu (M.J.C.-C.); s.arbe@tamu.edu (S.A.); stalcott@tamu.edu (S.T.T.); ndrury@cvm.tamu.edu (N.L.D.); ss91918@tamu.edu (S.S.)

**Keywords:** mango polyphenols, polyphenol metabolites, gut health, gut microbiota, intestinal integrity, inflammation

## Abstract

Mango is rich in polyphenols including gallotannins and gallic acid, among others. The bioavailability of mango polyphenols, especially polymeric gallotannins, is largely dependent on the intestinal microbiota, where the generation of absorbable metabolites depends on microbial enzymes. Mango polyphenols can favorably modulate bacteria associated with the production of bioactive gallotannin metabolites including *Lactobacillus plantarum*, resulting in intestinal health benefits. In several studies, the prebiotic effects of mango polyphenols and dietary fiber, their potential contribution to lower intestinal inflammation and promotion of intestinal integrity have been demonstrated. Additionally, polyphenols occurring in mango have some potential to interact with intestinal and less likely with hepatic enzymes or transporter systems. This review provides an overview of interactions of mango polyphenols with the intestinal microbiome, associated health benefits and underlying mechanisms.

## 1. Introduction

Mango (*Mangifera indica* L.) is a member of the *Anacardiaceae* family, which originated in India and traditionally grows in tropical climates [1]. Recently, mango production has increased worldwide. While specifically in the U.S., market saturation is limited, consumption has been steadily increasing [2]. Worldwide, there are over one thousand cultivars of mango. Alphonso is the most popular variety, native to India and rated the best in the world due to its aroma, delicious taste, and high nutritive value [3]. The cultivars that U.S. consumers are most likely to purchase are Tommy Atkins, Kent, Keitt, Haden, and Ataulfo [2,4]. Depending on the cultivar, mango varies in sensory properties (size, shape, weight, sweetness, and skin color) as well as nutritional and nutraceutical values [5]. Although the mango flesh is mainly consumed, since ancient times, the consumption of mango stem bark and leaves, mainly as infusions, has been reported, for pharmacological purposes and traditional medicine, especially in countries of Southeast Asia and Africa [6,7].

Recent research has demonstrated the relevance of mango polyphenols to intestinal health and in the prevention of chronic inflammatory diseases including inflammatory bowel diseases [8]. The intestinal microbiome plays a crucial role in building and maintaining intestinal barrier function, metabolizing unabsorbed food components, and regulating the intestinal immune system [9]. The use of probiotics and prebiotics modulate the composition of the gut microbiota to influence host immune response. Prebiotics such as fiber and non-digestible oligosaccharides are known to promote the growth of beneficial gut microbiota [10]. Several recent studies have shown the potential of polyphenol–microbiota interaction that polyphenols, including gallotannins rich in mango, act in the gut microbiota as prebiotics and the gut microbiota act on polyphenols to increase their bioavailability [11,12]. This review provides an overview of the most recent findings relevant to the impact of mango polyphenols on the intestinal microbiome and intestinal health.

## 2. Bioavailability and Intestinal Metabolism of Mango Polyphenols

Mango is an excellent source of polyphenols [13,14]. The polyphenolic profile in mango fruit is complex and it varies depending on the variety, physiological maturity stage, and the part of the fruit (peel, pulp, seed kernel) [15]. The primary polyphenols identified in mango pulp from different commercial varieties are gallic acid and galloyl-derived polyphenols, including mono-galloyl glucose, and gallotannins (hexa- to nona-*O*-galloyl-glucoses) and represent up to 95% of all polyphenols [16]. Gallic acid can be found in both free form or conjugated form (gallotannins), and it is considered the most prevalent monomeric polyphenol in mangoes [17]. Gallotannins are constituted of gallic acid linked to a polyol, prevalently glucose, but can also include glucitol, hamamelis, shikimic acid, quinic acid, and quercitol [18]. Minor polyphenols in mango pulp include vanillic, protocatechuic, p-hydroxybenzoic acid (hydroxybenzoic acid derivatives), p-coumaric, ferulic, cinnamic, caffeic, chlorogenic acid (hydroxycinnamic acid derivatives), mangiferin (xanthones), and quercetin derivatives (flavonoids) [17,19,20].

Mango peel is a promising source of polyphenols including mangiferin, a unique bioactive compound in mango [21], quercetin derivatives, rhamnetin, kaempferol [17], gallotannins [22], anthocyanins (cyanidin, peonidin, petunidin, delphinidin, and pelargonidin) [23], and proanthocyanidins (procyanidins A1, A2, A3, B2, B3) [20,24]. During ripening, mango cultivars change color to yellow or red. The yellow types have observed the higher carotenoid content, and the red types possess the higher anthocyanin content in the peel. The major compounds of anthocyanins in mango peel are cyanidin 3-*O*-galactoside, and peonidin 3-*O*-galactoside [25], and the levels of anthocyanins and proanthocyanidins in the peel tend to accumulate in higher concentrations in the later stages of maturation [24].

The realized health benefits of mango polyphenols rely on their bioaccessibility or bioavailability to the human body. The term bioaccessibility refers to the portion of a substance or an active compound readily available for absorption in the gastrointestinal system [26]. In contrast, bioavailability defines the portion of the compound that reaches the systemic circulation after oral absorption compared to systemic administration [27]. Several studies have shown that the bioaccessibility or bioavailability of polyphenols in mango fruit can be affected by variety, ripening stage, chemical structure, matrix interaction, and food processing [28,29,30]. The metabolism of polyphenols begins in the oral cavity with the hydrolysis of some glycosylated compounds through the action of salivary enzymes and oral bacteria [31]. Previous research has shown that larger tannins, especially gallotannins, can interact with salivary proteins, specifically proline-rich proteins [32,33]. A recent study by Sirven et al. showed that the interactions between salivary proteins and mango gallotannins during oral digestion reduce the bioaccessibility of gallic acid during the gastric phase of digestion [34]. Further chemical reactions such as hydrolysis and deglycosylation occur during gastric digestion and in the small intestine. In general, monomeric polyphenols are mainly absorbed in the small intestine [31], but polymeric polyphenols are not available for absorption in the small intestine [30,35]. Consequently, the non-absorbed portion of phenolic compounds reaches the large intestine and is metabolized by microbial enzymes [36]. Gallotannins are commonly found in a polymerized form, therefore their absorption is mainly influenced by enzymatic hydrolysis performed by gut microbiota. For the hydrolysis of gallotannins, a specific enzyme tannase produced by intestinal bacteria (tannin acyl hydrolase, EC 3.1.1.20) can hydrolyze the galloyl ester bonds of gallotannins to produce free gallic acid, and some bacterial species express gallate-decarboxylase (EC 4.1.1.59) to produce pyrogallol by decarboxylating gallic acid. Some intestinal bacteria belonging to Firmicutes phylum, including *Lactobacillus plantarum* and *Streptococcus gallolyticus*, produce tannin acyl hydrolase and gallate decarboxylase and are therefore able to metabolize gallotannins into gallic acid, pyrogallol, and eventually catechol [37]. After intestinal absorption, circulating gallotannin metabolites are found in plasma in glucuronidated, methylated, and sulfated forms. The urinary and biliary excretion rate for these compounds is quick because of their anionic properties [36]. The effective intestinal microbial metabolism of gallotannins results in significant amounts of gallotannin and pyrogallol metabolites in plasma and urine after mango consumption, where gallic acid derived from gallotannins and pyrogallol are proposed to be absorbed via enterohepatic circulation [38]. Barnes et al. identified seven gallic acid phase II metabolites in urine, from which pyrogallol-*O*-sulfate and deoxypyrogallol-*O*-sulfate were detected in significantly higher concentrations after 10 days of daily mango consumption (cv. Ataulfo 400 g), although pyrogallol was not detected in mango pulp [38]. Furthermore, in another human clinical study, Barnes et al. identified five gallotannin metabolites (4-*O*-methylgalic acid, 4-*O*-methylgalic acid-3-*O*-sulfate, pyrogallol-*O*-sulfate, methylpyrogallol-*O*-sulfate, and catechol-*O*-sulfate) in plasma and urine. These results demonstrated the absorption of these metabolites in the colon into plasma and urinary excretion within 24 h of ingestion [39].

The interaction between polyphenols and macronutrients within the food matrix is another factor that influences the bioavailability of mango polyphenols. It had previously been observed that dietary fiber affects the absorption of phenolic compounds in the small intestine [35]. Consequently, the application of processing techniques such as heat treatments, grinding, fermentation, and homogenization may improve the bioaccessibility and bioavailability of bioactive compounds from complex matrices [40]. Quirós-Sauceda et al. investigated the matrix effect (juice vs. flesh) on the bioavailability of phenolic compounds from mango (var. Ataulfo) in humans. They detected five phenolic acids, including gallic, chlorogenic, ferulic, protocatechuic, and gentisic acid in plasma, with a C_max_ at 2–4 h after mango consumption. Similarly, in urine samples, six phenolic acids were recovered. The group that consumed mango juice presented higher concentrations of gentisic, chlorogenic and ferulic acids in plasma and p-coumaric and ferulic acids in urine compared to the pulp. These findings suggested that mango pulp processing into juice may improve polyphenolic compounds’ bioavailability [41].

## 3. Interactions between Mango Polyphenols and the Intestinal Microbiota

The intestinal microbiota is estimated to be composed of more than 100 trillion microbial cells residing within the gastrointestinal tract [42] and exerts a key role in the metabolism and bioavailability of polyphenols; thus, inter-individual differences in gut microbiota composition could influence the bioactivity of polyphenols and their metabolites as well as the risk of metabolic disorders [12,43]. It is reported that microbial species such as *Bifidobacterium* spp., *Lactobacillus* spp., *Akkermansia* spp., *Bacteroides* spp., and *Eubacterium* spp. produce microbial enzymes (e.g., esterase, glucosidase, demethylation, dihydroxylation, and decarboxylation) that can metabolize polymeric polyphenols into low-molecular metabolites that are better absorbed than the parent compound [44,45]. Additionally, several polymeric polyphenols can interact with the surface proteins of gut microbiota to affect their activity and have antibacterial activity [46]. Multiple *in vitro*, animal, and human intervention studies have evaluated the impact of mango polyphenols on gut microbial composition. Additionally, the concentration of dietary fiber in mango and mango by-products provides a considerable number of polysaccharides (cellulose/pectin) that can be partially or completely fermented by the gut microbiota. Overall, mango supplements may have a significant impact on the intestinal microbial composition (Table 1).

### 3.1. Modulation of Pathogenic Bacteria by Mango Polyphenols

Mango polyphenols were investigated in their potential antimicrobial properties on intestinal pathogenic bacteria. Several studies have reported that polyphenols distributed in several concentrations in different parts of mangoes like kernel, peel, and pulp are responsible for their purported antimicrobial properties [53]. In general, mango polyphenols have shown greater inhibition against Gram-positive bacteria than Gram-negative bacteria, explained by the structural differences in their cell walls. Gram-positive bacteria are more vulnerable to having an altered permeability in the microbial membrane. For example, Mutua et al. [54], reported that mango kernel extracts exerted a greater inhibition against *Streptococcus aureus* than *Escherichia coli*, and these results were attributed to tannins and flavonoids in the extract. Similarly, Engels et al. showed that gallotannins extracted from mango kernels had greater antimicrobial activity against *Bacillus subtilis* and *S. aureus* compared to *E. coli* [55]. Interestingly, these effects occurred without inhibiting the growth of beneficial lactic acid bacteria, suggesting the selective inhibitory activity of mango gallotannins, likely based on the toxic nature of gallotannins to bacteria that do not possess the ability to metabolize these compounds [45], where gallotannins and even gallic acid can entangle surface proteins on bacteria, particularly those rich in proline and that cause dysfunction [56,57]. Beneficial lactic acid bacteria that can produce tannase are expected to be able to break down gallotannins and prevent crosslinks of their surface proteins.

Gallic acid has been reported to exert inhibitory effects against intestinal pathogenic bacteria. For instance, *Clostridium histolyticum*, a pathogenic bacterium associated with an increased risk of developing cancer and inflammatory bowel disease, was inhibited by gallic acid [58]. Likewise, gallic acid derived from tea extracts also repressed the levels of *Clostridium perfringens*, *Clostridium difficile*, and *Bacteroides* spp., which are risk markers for colon cancer [59]. Interestingly, Lee et al. reported the selective inhibitory effects of gallic acid as this phenolic compound inhibited the growth of pathogenic *Clostridium* spp., while commensal *Clostridium* spp. were enhanced [59]. Similarly to tea, mango is also rich in gallic acid and is likely to show the same inhibitory effects against intestinal pathogenic bacteria.

### 3.2. Modulation of Probiotic Bacteria by Mango Polyphenols

While the term probiotic has not yet been officially regulated, it is used in this review to refer to beneficial bacteria [60]. Mango polyphenols can modulate the composition and activity of probiotic gut bacteria conferring multiple health benefits to the host. In addition, further evidence has associated the dietary fiber concentration in mangoes with prebiotic effects. Several studies have assessed the effects of mango polyphenols under dysbiotic conditions as they occur in obesity and intestinal chronic diseases [61]. Mango polyphenols were investigated for their potential to restore the balanced composition of gut microbiota. Freeze-dried mango pulp modulated the gut microbiota composition in mice fed with a high-fat diet, in an increase in the beneficial *Bifidobacteria* spp. and *Akkermansia muciniphila* and enhanced short-chain fatty acid (SCFAs) production [50]. *Bifidobacteria* spp. are known for their probiotic effects and for promoting intestinal homeostasis by enhancing gut barrier function, whereas *A. muciniphila* has been inversely correlated with obesity-inflammatory conditions. Similarly, our group has previously reported that 6-week mango consumption increased the levels of tannase-producing *Lactococcus lactis* and reduced the abundance of bacteria associated with obesity such as *Bacteroides thetaiotaomicron* and *Clostridium leptum* to resemble the levels observed in lean participants [39].

In our previous study, the anti-inflammatory activities of mango polyphenols on intestinal microbiota composition were evaluated in rats with dextran sulfate sodium (DSS)-induced colitis. Results indicated that that the intake of mango polyphenols intake (at 476 mg gallic acid equivalents/L) increased the abundance of tannase-producing bacteria *Lactobacillus plantarum* and *Lactococcus lactis*, and butyrate-producing bacteria *Clostridium butyrium*, and these beneficial effects were accompanied by the enhanced production of SCFAs butyrate and valerate [49]. *L. plantarum* and *L. lactis* are known to produce enzymes to hydrolyze gallotannins to gallic acid, pyrogallol and catechol [62], which were reported to exert anti-inflammatory activity in the prevention and treatment of colitis [63]. *C. butyrium* has also demonstrated anti-inflammatory effects due to the production of butyrate [64], which is an essential metabolite known to promote gut barrier function and decrease inflammation [65]. Likewise, our group has previously reported that mango supplementation (200 to 400 g) for 8 weeks may be effective as an adjuvant in combination with conventional medications to treat gut dysbiosis in IBD patients. The intake of mango pulp has significantly increased the relative abundance of *L. plantarum*, *L. reuteri*, and *L*. *lactis* and enhanced the production of butyric acid [51]. Overall, findings from these studies indicate that mango polyphenols can have beneficial effects on intestinal health by favorably regulating bacteria involved in the production of bioactive gallotannin metabolites.

Even though the possibility of polyphenols as modulators of the gut microbiota is being studied, the dosing, dosing frequency, and their interactions with dietary fiber have not been established with certainty [66]. Mangoes are also a rich source of dietary fiber, which can serve as substrates for bacterial fermentation. Dietary fiber is distributed in the different parts of the mango including pulp and peel. Sayago et al. [47] suggested the prebiotic potential of mango (var. Ataulfo) peel, as the levels of *Bifidobacterium*, *Lactobacillus*, *Dorea*, and *Lactococcus* were increased after 72 h of in vitro colonic fermentation. In a later study, Gutierrez-Sarmiento et al. [48], analyzed the effects of the indigestible fraction from a mango-based bar (Ataulfo var.) on the gut microbiota composition through an in vitro gut fermentation model. Findings from this study showed that the levels of *Faecalibacterium*, *Roseburia*, *Eubacterium*, and *Bifidobacterium* among other genera were increased after 6 h of colonic fermentation as assessed by 16 s rRNA sequencing. This modulation is relevant because *Faecalibacterium*, *Roseburia*, and *Eubacterium* are known as butyrate producers. In addition, emerging studies have demonstrated that ulcerative colitis patients have a predominant decrease in butyrate-producing bacteria such as *Roseburia hominis* and *Faecalibacterium prausnitzii* [67], but the consumption of mango bars partially restored the levels of these bacteria. Overall, these findings indicate that the consumption of mango is likely to exert prebiotic effects, and this, in turn, will contribute to gut integrity and intestinal homeostasis. In addition, the growth stimulation of probiotic bacteria is beneficial for the host due to the increased production of SCFAs, which are used by the cells as a source of energy.

The intestinal microbiota and produced metabolites produced from polyphenolic compounds largely impact the gut–brain axis. The gut–brain axis describes the bidirectional connection between the cognitive and emotional centers of the brain with that of the peripheral intestine through the vagus nerve of the central nervous system [68]. The *Bifidobacterium*, *Lactobacillus plantarum*, and *Akkermansia muciniphila* associated with gallotannin metabolism within the gut has been described by others to increase serotonin signaling [68,69,70]. *Bacteroides thetaiotaomicron* is attributed to the motility of the gastrointestinal tract [71]. Butyrate producers, such as *Eubacterium*, contribute to the SCFAs pool and the modification of various signaling pathways involving G-protein-coupled receptors and the inhibition of histone deacetylases [72]. Furthermore, many polyphenolic compounds of *Mangifera indica* and metabolites thereof have been described to improve the cognitive function of learning and memory function as well as areas of cognitive impairment [73,74]. Specifically, gallic acid—the product of gallotannin hydrolysis and mangiferin, a c-glucosyxanthone (C2-β-d-glucopyranosyl-1,3,6,7-tetrahydroxyxanthone) capable of crossing the blood–brain barrier—has been the main target of these studies. In a diabetic mouse model, Infante-Garcia et al. found a decrease in tau phosphorylation, a common pathological phenomenon in Alzheimer’s disease, when mango leaf extract was administered to db/db mice which limited cortical and hippocampal atrophy [73]. Similarly, Yu et al. found that gallic acid disrupted Aβ aggregation which in turn alleviated cognitive decline in a model for Alzheimer’s disease using transgenic APP/PS1 mice [75]. Gallic acid also protected against cognitive decline induced by chronic cerebral hypoperfusion and oxidative damage [76].

Overall, mango polyphenols may reach the lower intestinal tract and exert a favorable modulation of the gut microbiota. Mango supplementation often resulted in the restoration of dysbiotic intestinal microbiota, which may also affect the gut–brain axis and cerebral function and diseases. The modulation of the gut microbiota by mango polyphenols and dietary fiber is relevant given the key role that the gut microbiota exerts for metabolic, nutritional, physiological, immunological, and cognitive processes [77].

## 4. Mango Polyphenols in Gastrointestinal Disorders

Mango polyphenols have been shown to exhibit various pharmacological activities such as antioxidant, anti-bacterial, anti-inflammatory, gastroprotective, immunomodulatory, and cancer-cytotoxic effects [6,78,79,80,81]. The anti-inflammatory activities of gallotannins and associated metabolites, gallic acid and 4-*O*-methylgallic acid mediated via the reduction in proinflammatory cytokines, nuclear factor kappa B (NF-κB), intracellular adhesion molecule 1 (ICAM-1), and vascular cell adhesion molecule 1 (VCAM-1) have been well-reviewed [82]. Gallotannins may modulate the immune system of the gastrointestinal tract mediated by the production of metabolites and the modulation of the intestinal microbiome.

Inflammatory bowel disease (IBD) is a group of gut disorders (Crohn’s disease and ulcerative colitis) that are characterized by the chronic inflammation of the digestive tract and an increased risk of colorectal cancer [83]. The intestinal microbiome is crucial in maintaining colon health via several mechanisms [84], and microbial dysbiosis is one of the factors linked to the progression of IBD [85]. Due to the side effects of conventional treatments, the use of dietary polyphenols has become an attractive approach for treating IBD due to their pharmaceutical effects. Additionally, the beneficial modulation of the gut microbiota by mango consumption shows the possibility of being effective on intestinal inflammation.

Intestinal epithelial cells play important roles in the mucosal inflammatory response, including providing a barrier, maintaining gut structural integrity, modulating gut microbiota, and producing cellular events (proliferation and apoptosis) and inflammatory mediators such as cytokines and chemokines [86]. Caco-2 in vitro intestinal model is used to evaluate the anti-inflammatory effects of polyphenols in intestinal epithelial cells [86], and in these experiments, mango polyphenols showed the potential on intestinal epithelial cells to reduce the inflammation responses by modulating pro-inflammatory cytokines. Gallic acid inhibited inflammation via a reduction in proinflammatory cytokines including interleukin-1 (IL-1), interleukin-6 (IL-6), interleukin-17 (IL-17), and tumor necrosis factor alpha (TNF-α) and an induction in anti-inflammatory cytokines including interleukin-4 and interleukin-10 (IL-4 and IL-10), and induced apoptosis via the inhibition of NF-κB in an inflamed HIEC-6 model [87]. In addition, gallotannins mixed with gelatin showed anti-inflammatory properties through the inhibition of ICAM-1, interleukin-8 (IL-8), and TNF-α in inflamed Caco-2 cells [88]. Mango peels are promising sources of polyphenols containing mangiferin, proanthocyanidins, and anthocyanins, which have pharmacological effects including antioxidant, anticancer, and anti-inflammatory properties [20,89]. Proanthocyanidins, also known as condensed tannins, are polymeric polyphenols of catechins (flavan-3-ols) [90], which also show prebiotic properties that are catabolized by gut microbiota [11]. The proanthocyanidin fraction from pistachio showed anti-inflammation properties in inflamed Caco-2 cells via the suppression of prostaglandin E2 (PGE2), IL-6, IL-8, cyclooxygenase-2 (COX-2), and NF-kB, and inhibited the increased paracellular permeability [91]. In addition, proanthocyanidin rich-grape seed extract showed similar effects with reducing oxidative stress and increasing epithelial barrier integrity to improve gut health in inflamed Caco-2 cells [90]. Anthocyanins-rich extract from wild blueberry displayed anti-inflammatory properties by decreasing the activation of NF-κB in Caco-2 cells [92]. These results show the effects of gallotannin metabolites as well as polyphenols in mango peels on intestinal epithelial cells to improve gut health. Additionally, mango byproducts, especially peels, are considered for industrial use due to their pharmaceutical properties [90].

The anti-inflammatory effects of mango extracts and their compounds (gallic acid, mangiferin) have been studied in colitis-induced murine models. In our previous studies, the intake of mango polyphenols improved intestinal histology-scores in DSS-treated rats. It also decreased the protein-levels of the pro-inflammatory cytokines, C-reactive protein (CRP), TNF-α, interleukin 1 beta (IL-1β), granulocyte monocyte cell stimulating factor (GM-CSF), and IL-6 and attenuated the colitis symptoms by the modulation of the phosphoinositide 3-kinase/protein kinase B/mechanistic target of rapamycin (PI3K/AKT/mTOR) pathway via the up-regulation of the miR-126 and histone deacetylase/AMP-activated protein kinase (HDAC1/AMPK) pathway [49,93]. In addition, an in silico molecular docking study indicated a high binding of gallic acid to the catalytic domain of insulin-like growth factor type 1 receptor (IGF-1R), which may suppress the AKT/mTOR axis. Fecal isovalerate and valerate SCFA-production was increased by mango polyphenols and may be at least in part involved in their anti-inflammatory effects [94]. In other studies, mango extract showed anti-inflammatory activities through the reduction in inducible nitric oxide synthase (iNOS), COX-2, TNF-α, and TNF receptor-2 (TNFR-2) in the same DSS models [80]. Vimang, a stem bark extract of the mango, also showed anti-inflammatory activity through the inhibition of PGE2 [79], TNF, and nitric oxide (NO) in the experimental colitis model, in vivo and in vitro [95]. Mangiferin, a unique bioactive compound in mango [21], was shown to attenuate inflammation in mice colitis models through the suppression of NF-κB and mitogen-activated protein kinase (MAPK) signaling [96] or the correction of the imbalance of Th17/Treg cells [97]. In the molecular docking model, the structure of mangiferin showed a high binding potential with TNF-α and MMP-9 and a reduced colonic injury partly through attenuating the activity of TNF-α and MMP-9 in the colitis model [98]. In our pilot study, 8-week consumption of mango (200–400 g) significantly improved the Simple Clinical Colitis Activity Index (SCCAI) score in participants with IBD and decreased the levels of pro-inflammatory cytokines including IL-8, growth-related oncogene (GRO), and GM-CSF, factors that are related to neutrophil filtration [51]. These results show that gallotannin-rich mango preparations may positively contribute to the long-term management of IBD.

Colon cancer is one of the most common cancers in the US [99]. Mango has been shown to have the potential to prevent colon cancer in several in vitro studies. Our previous study shows that two varieties—Ataulfo and Haden—were the most effective in reducing cell growth via cell cycle arrest and decreased reactive oxygen species (ROS) production in SW-480 colon carcinoma cells when compared to polyphenol extracts from other varieties Kent, Francis, and Tommy Atkins [81]. The treatment of Ataulfo mango peel polyphenols showed the highest antioxidant and antiproliferative activities in LS180 human colon adenocarcinoma cells [100]. Additionally, mango peel extracts reduced cell viability via γH2AX-mediated apoptosis associated with ROS induction, the activation of c-Jun N-terminal kinase (JNK), extracellular signal-regulated protein kinase 1 and 2 (ERK1/2), manganese superoxide dismutase (MnSOD), and nuclear factor erythroid 2-related factor 2 (Nrf2) in human colorectal cancer cell lines (Caco-2, HCT116, and HT-29) [101]. In mice, mango pulp intake reduced the formation of aberrant crypt in AOM-treated mice [102], but the underlying mechanism was not demonstrated.

In intestinal health, it is crucial to maintain intestinal integrity through tight junction proteins, including occludin, claudins, and zonula occludens. Increased intestinal permeability is observed in several intestinal chronic diseases, including IBD and colon cancer, and correlated with an increased level of inflammation. Intestinal inflammation caused barrier breakdown leading to the inflammation of the intestinal mucosa and impairment of integrity, allowing pathogenic bacteria, toxins, and antigens from the lumen to translocate into the bloodstream and cause several dysfunctional conditions [103]. Several studies have reported that polyphenols have the potential to modulate intestinal permeability [104], and although not directly tested with polyphenols from mango, the intake of gallotannins was shown to improve intestinal barrier integrity and function through the increase in zonula occludens-1 (ZO-1) mRNA expression in oxidative stress induced in a mouse model [105]. In another study of weaning piglets with a high frequency of diarrhea due to impaired tight junctions, tannic acid intake reduced diarrhea by up-regulating the expression of occludin and zonula occudens-2 (ZO-2) mRNA [106]. Hence, mango polyphenols may contribute to the maintenance of tight junctions of intestinal epithelial cells and strengthen intestinal integrity.

We previously reported that mango intake showed a beneficial effect on constipation. The 4-week consumption of mango fruit (300 g) in a human clinical pilot study significantly improved the status of constipation (stool frequency, consistency, and shape) and increased gastrin levels and intestinal valeric acid, while lowering endotoxin and IL-6 in comparison to an equivalent amount of dietary fiber [107].

These studies show that mango polyphenols have the potential to maintain and improve intestinal health and integrity.

## 5. Potential Interactions of Mango Polyphenols with Intestinal and Hepatic Enzyme Systems

In addition to the aforementioned diverse beneficial health benefits, polyphenols have been described to interact with intestinal and hepatic enzyme-systems. Specifically, effects on the cytochrome P450 (CYP) enzyme system, and how these interactions affect xenobiotic metabolism, are of particular concern although evidence is limited. Although there are over 50 different CYP enzymes, six are responsible for > 90% of drug metabolism, with the two most important enzymes being CYP3A4 and CYP2D6 [108]. The CYP enzymes have diverse expressions throughout the different regions of the gut, and thus the CYP profile in the gut is difficult to characterize. CYPs are mainly active in the liver but have some activity in the small intestine and the colon since both sites serve as absorbing and metabolizing organs [109].

An important aspect of the cytochrome P450 (CYP) is that there is a crosstalk axis between enzyme expression and activity, and the gut microbiota. An interference with the CYP system could affect the intestinal microbiome and vice versa [110]. Modifiers of the intestinal microbiota, such as probiotics, may alter the expression of drug-metabolizing enzymes in the liver, affecting xenobiotic metabolism [111]. Some polyphenols also found in mango have been documented to interfere with or affect the functioning of the CYP system. In vitro studies have described that isolated mangiferin or a mango stem bark extract (MSBE; containing mangiferin, quercetin, gallic acid, and catechins) can inhibit the activities of CYP3A4 in primary human hepatocytes; however, these effects were observed at µg/mL levels, which starkly contrasts with the typical concentrations expected to be found within the gut [112,113,114,115,116]. Hence, it is uncertain whether described interactions are to be considered clinically relevant. Metabolic studies indicate that interaction with the CYP3A enzymes primarily occurs in the intestine rather than exclusively in the liver where polyphenol concentrations are much lower compared to the intestines [117]. In Crohn’s Disease or IBD, intestinal levels of CYP3A4 can be lower [118]. CYP3A4 is important for the metabolism of drugs such as cyclosporin, an immunosuppressant or budesonide is a common corticosteroid that may be prescribed to patients with IBD. For this reason, an acute high dose of polyphenols may interfere with drug metabolism in a situation where CYP-enzyme levels are already decreased [119]. CYP2E1 is also a target that can be inhibited by polyphenols (e.g., quercetin) that occurs in mangos in low concentrations [120,121]. CYP2E1 activity is typically only exerted in the liver, where circulating polyphenol concentrations are very low; hence, this interaction is not likely to be clinically relevant. Clinically relevant enzyme interactions might be relevant in severe pathological situations [119].

In contrast, the inhibition of CYP-metabolism by polyphenols may reduce the severity of toxic exposures. Toxic exposures are of particular concern in areas of poverty, where the access to clean water or adequate food sources is limited [122]. For example, aflatoxin B1 (AFB1) can be found in contaminated drinking water and grains. CYP3A4 is involved in activating the toxic derivative of AFB1 to the toxic 8,9-*exo*-epoxide metabolite. Reducing CYP3A4-activity will reduce the production of this toxin [123]. Similarly, CYP2E1 is involved in the conversion of carbon tetrachloride (CCl4), used as refrigerant or dry-cleaning agent, to reactive, free-radical intermediates, possibly inducing damage to hepatic mitochondria; carbon tetrachloride can also contaminate water and soil sources, inducing potential exposures to target populations [124]. In this case, the inhibition of CYP2E1 might be beneficial. Before being alerted by any CYP-polyphenol interaction that has been demonstrated in vitro or animal studies with high concentrations of study treatments, it is crucial to evaluate physiologically relevant polyphenol levels at the respective target site as well as drug or toxicant concentrations in real-life situations.

## 6. Conclusions

**Figure 1:**
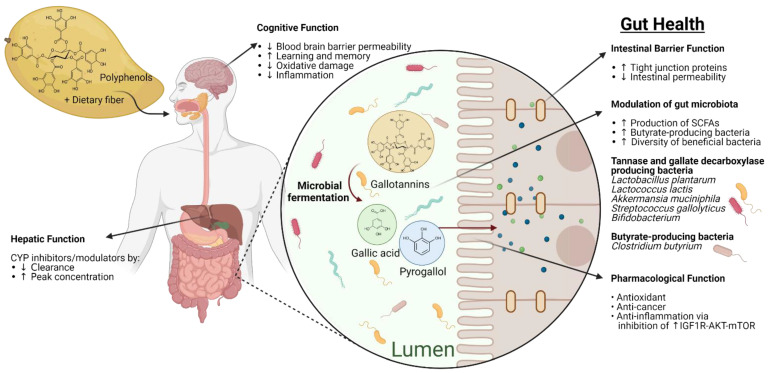
Overview of the proposed mechanism of actions of mango polyphenols and fiber consumption on human intestinal health. Mango polyphenols and fiber may promote gut health through their pharmacological activities, modulation of beneficial gut microbiota (tannase, gallate decarboxylase, and butyrate-producing bacteria) and thus, contribute to maintaining the gut barrier and cognitive function. Created using BioRender (https://biorender.com/, accessed on 15 April 2021) as part of Academic License.

Mango is rich in polyphenols, predominantly gallic acid, and gallotannins conveying pharmacological activities, including antioxidant, anticancer, and anti-inflammatory activities that have been studied in improving resilience against chronic inflammatory diseases in the intestines or improving chronic inflammation. The bioavailability of polyphenols derived from polymers such as gallotannins depends on the metabolism by the intestinal microbiota. Polyphenols, as well as dietary fiber from mango, may serve as prebiotics to increase probiotic bacteria in the intestines. In addition, the anti-inflammatory effect of mango has been demonstrated to prevent or mitigate inflammation and other symptoms associated with chronic intestinal diseases, colon cancer, leaky intestines, and constipation via the suppression of pro-inflammatory cytokines, as well as improve intestinal health. The interaction of mango polyphenols with intestinal and hepatic enzyme-systems is possible but unlikely with dietary intake levels. Recently, studies on the gut–brain axis investigated the influence of polyphenols occurring in mango on cognitive function via the gut–brain axis (Figure 1). Further studies need to investigate dose levels, inter-individual variability, and pharmacometrics aspects of mango polyphenols on a pathway to quantitative intake recommendations in the improvement or maintenance of intestinal health.

## Figures and Tables

**Table 1:** Gut microbiota modulation by mango polyphenols, summarizing changes in short chain fatty acids (SCFA).

Parts of Mango Fruit	Type of Study	Dose	Length of Treatment	Gut Microbiota Modulation	Changes in SCFAs	Ref.
Pulp (cv. Ataulfo)	Human pilot trial (lean and obese subjects)	400 g/daily	6 weeks	Obese: Increased levels of *Lactococcus lactis* and decreased levels of *Clostridium leptum* and *Bacteroides thetaiotaomicron* Lean: no significant changes	Lean: increased trend in butyric and valeric acid fecal levels	[39]
Peel (cv. Ataulfo)	In vitro model of human colon (TIM-2)	7.5 g	0, 24, 48, and 72 h	Increased levels of *Bifidobacterium* and *Lactobacillus* at 24 h	No significant changes in SCFA production	[47]
Pulp and peel (cv. Ataulfo)	In vitro colonic fermentation	500 mg mango bar (snack)	0, 6, 12, 24, and 48 h	Induced growth of *Faecalibacterium*, *Roseburia*, *Eubacterium*, *Fusicatenibacter*, *Holdemanella*, *Catenibacterium*, *Phascolarctobacterium*, *Buttiauxella*, *Bifidobacterium*, *Collinsella*, *Prevotella*, and *Bacteroides* genera Increased Bacteroidetes phylum and decreased Firmicutes/Bacteroidetes ratio.	Enhanced production of acetic acid in 30 gr of mango bar with 9.5% dietary fiber (DF)	[48]
Pulp (cv. Keitt)	Animal study (rats with DSS-induced colitis)	Ad libitum beverage (89.74 mg GAE/kg/d)	9 weeks	Significant increase in *Lactobacillus plantarum* and *Lactococcus lactis*, and *Clostridium butyrium*	Increased production of butyric and valeric acids	[49]
Pulp (cv. Tommy Aktins)	Animal study (mice fed a high-fat diet)	1% or 10% freeze-dried mango	12 weeks	Favorable modulation of *Bifidobacteria* and *Akkermansia muciniphila*	Increased levels of fecal acetic and butyric acids.	[50]
Pulp (cv. Keitt)	Human pilot trial (IBD subjects)	200 to 400 g/d	8 weeks	Significant increase in the abundance of *Lactobacillus plantarum*, *Lactobacillus reuteri*, and *Lactobacillus lactis*	Increased fecal butyric acid production	[51]
Pulp	Animal study (pigs, healthy)	15% dried mango pulp	3 weeks	Increased levels of *Faecalibacterium prausnitzii*	Increased trend in total SCFAs	[52]
